# Identification of MicroRNA Targets of *Capsicum* spp. Using MiRTrans—a Trans-Omics Approach

**DOI:** 10.3389/fpls.2017.00495

**Published:** 2017-04-10

**Authors:** Lu Zhang, Cheng Qin, Junpu Mei, Xiaocui Chen, Zhiming Wu, Xirong Luo, Jiaowen Cheng, Xiangqun Tang, Kailin Hu, Shuai C. Li

**Affiliations:** ^1^Department of Computer Science, City University of Hong KongHong Kong, China; ^2^Pepper Institute, Zunyi Academy of Agricultural SciencesZunyi, China; ^3^Guizhou Provincial College-based Key Lab for Tumor Prevention and Treatment with Distinctive Medicines, Zunyi Medical UniversityZunyi, China; ^4^BGI-ShenzhenShenzhen, China; ^5^College of Horticulture and Landscape Architecture, Zhongkai University of Agriculture and EngineeringGuangzhou, China; ^6^College of Horticulture, South China Agricultural UniversityGuangzhou, China

**Keywords:** pepper (Capsicum spp.), miRNA targets, miRNA sequencing, transcriptome sequencing, degradome sequencing, lasso regression

## Abstract

The microRNA (miRNA) can regulate the transcripts that are involved in eukaryotic cell proliferation, differentiation, and metabolism. Especially for plants, our understanding of miRNA targets, is still limited. Early attempts of prediction on sequence alignments have been plagued by enormous false positives. It is helpful to improve target prediction specificity by incorporating the other data sources such as the dependency between miRNA and transcript expression or even cleaved transcripts by miRNA regulations, which are referred to as trans-omics data. In this paper, we developed MiRTrans (Prediction of MiRNA targets by Trans-omics data) to explore miRNA targets by incorporating miRNA sequencing, transcriptome sequencing, and degradome sequencing. MiRTrans consisted of three major steps. First, the target transcripts of miRNAs were predicted by scrutinizing their sequence characteristics and collected as an initial potential targets pool. Second, false positive targets were eliminated if the expression of miRNA and its targets were weakly correlated by lasso regression. Third, degradome sequencing was utilized to capture the miRNA targets by examining the cleaved transcripts that regulated by miRNAs. Finally, the predicted targets from the second and third step were combined by Fisher's combination test. MiRTrans was applied to identify the miRNA targets for *Capsicum* spp. (i.e., pepper). It can generate more functional miRNA targets than sequence-based predictions by evaluating functional enrichment. MiRTrans identified 58 miRNA-transcript pairs with high confidence from 18 miRNA families conserved in eudicots. Most of these targets were transcription factors; this lent support to the role of miRNA as key regulator in pepper. To our best knowledge, this work is the first attempt to investigate the miRNA targets of pepper, as well as their regulatory networks. Surprisingly, only a small proportion of miRNA-transcript pairs were shared between degradome sequencing and expression dependency predictions, suggesting that miRNA targets predicted by a single technology alone may be prone to report false negatives.

## Introduction

MicroRNAs (miRNAs) are small non-coding RNAs (~22-nt) that arise from short stem-loop precursors through the double-stranded ribonuclease (Bernstein et al., 2001). They are found widely exist in plants, animals, and bacteria. By directly binding to the transcripts, their regulations result in transcript cleavage or translational protein repression. The miRNAs serve as key regulators in cell proliferation, differentiation, metabolism, and apoptosis (Calin and Croce, 2006; Ameres and Zamore, 2013).

Although novel miRNAs remains to be discovered, exploring their targets is more crucial to understand the mechanisms of miRNA. Comparing with transcripts, miRNAs are of shorter lengths and they are supposed to bind their targets anywhere in the sequence. Many studies have been proposed to identify miRNA targets by sequence-based prediction tools (Zhang, 2005; Zhang et al., 2009; Dai and Zhao, 2011; Milev et al., 2011; Iossifov et al., 2014).

Sequence complementarity forms the basis of many miRNA target discovery tools. In plants, sequence complementarity is strongly observed, especially for the positions from the 2nd to the 13th of mature miRNA sequences (5′ end) with their targets (Dai and Zhao, 2011; Dai et al., 2011). Other characteristics of miRNA base-pairing were also exploited, e.g., target accessibility and the presence of multiple miRNA binding sites on the same transcript (Axtell et al., 2006; Brodersen and Voinnet, 2009). The free energy of sequence hybridization was also considered as an attribute of miRNA target prediction. The lower free energy required for binding, the more likely the binding site exists (Yue et al., 2009). RNAhybrid (Muckstein et al., 2006) was developed to utilize the free energy of hybridization, which successfully identified several novel targets of bantam in *Drosophila*, such as the *hid, Nerfin-1*, and *Dll* (Muckstein et al., 2006; Kertesz et al., 2007).

MiRNA targets predicted solely on sequence characteristics are glutted by false positives (Yue et al., 2009) since the sequences of miRNA-target pairs may be complementary by random chance. This may result in unproductive experimental validation. Alternative methods have been devised to eliminate false positives by investigating the expression correlations between miRNAs and their putative targets. The putative targets are removed if their expressions are weakly correlated with the miRNAs. These correlations are commonly computed by linear correlation (Xiao et al., 2009) or mutual information (Hsu et al., 2011). However, these two methods are unable to distinguish those false targets which co-express with the true ones. This issue can be alleviated by the predictive models such as multivariate linear regression (Jayaswal et al., 2009; Beck et al., 2011), regularized least square, and Bayesian network (Mootha et al., 2003; Carmona-Saez et al., 2007; Ragan et al., 2011). We applied transcriptome sequencing to quantify transcript expression, which was proved to be better than microarray (Iancu et al., 2012). On the other hand, degradome sequencing can help in identifying cleaved transcripts directly by sequencing the 5' ends of uncapped RNAs, which is considered as the miRNA regulation productions. Through this technology, Addo-Quaye et al. detected 100 potential miRNA targets in *Arabidopsis* (Addo-Quaye et al., 2008), which has been identified previously (Jones-Rhoades et al., 2006). Degradome sequencing also led to the identification of 160 targets of 53 miRNA families in rice (Li et al., 2010), including the *CCS1*, a novel and conserved target of miR398.

Most of the targets of a given miRNA are expected to be involved in the similar functions, especially for plant miRNAs. For instance, the targets of a human miRNA i.e., let-7b, are enriched in the genes (including *PRDM2, DUSO9, OSMR, NDST2*) from the same Gene Ontology annotation (Huang et al., 2007). This assumption allows one to refine miRNA targets by leveraging gene co-expression rank, as demonstrated in CoMeTa (Gennarino et al., 2012). Furthermore, for plants such as *Arabidopsis*, miRNA demonstrate a tendency to regulate genes from the same protein family. For example, miR156 and miR157 bind to the genes in Squamosa-promoter Binding Protein(SBP)-like proteins; the targets of miR170 and miR171 are enriched in GRAS domain proteins (SCARECROW-like) (Rhoades et al., 2002; Jones-Rhoades et al., 2006; Chen, 2009; Borges et al., 2011; Song et al., 2011).

**Figure 1:**
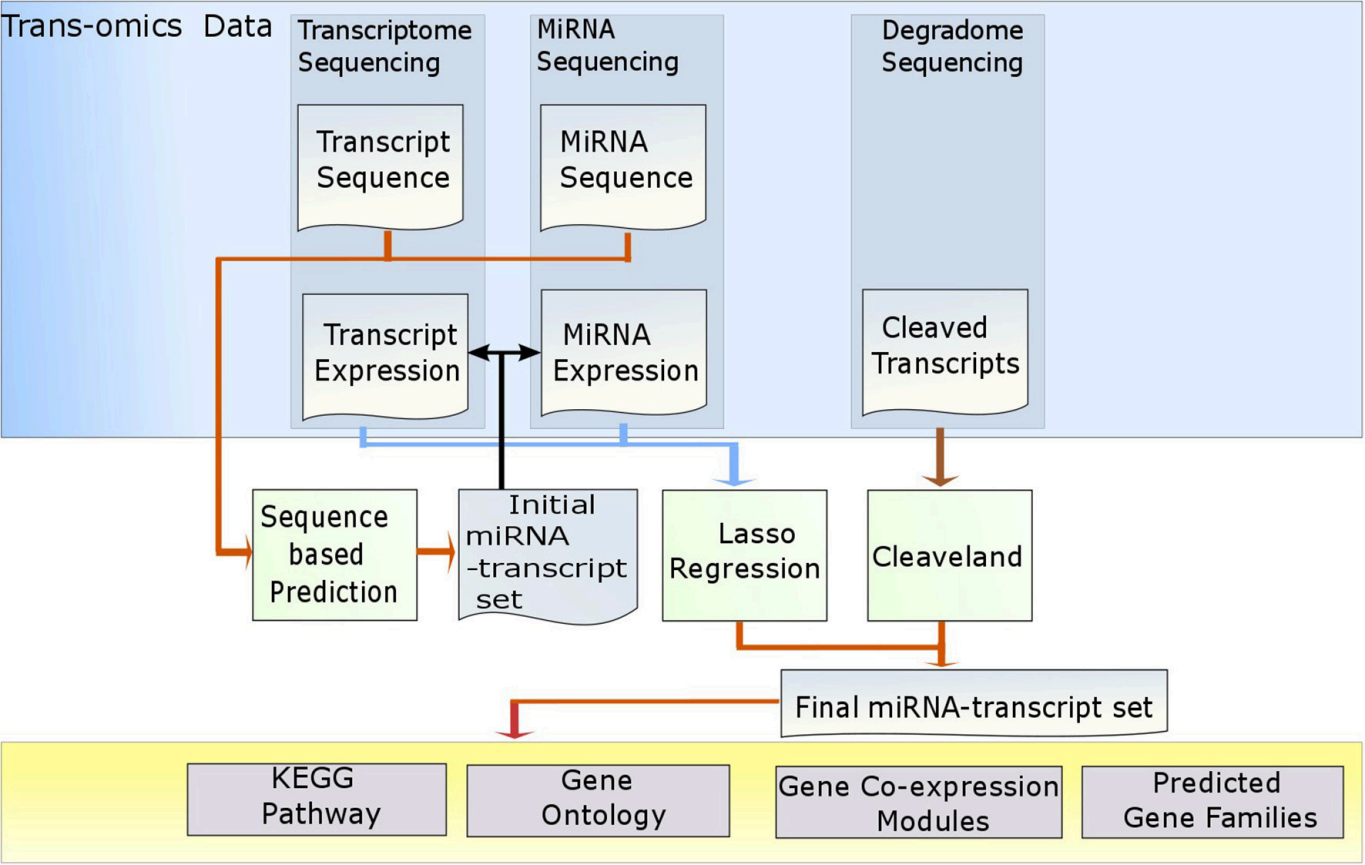
**The workflow of MiRTrans**.

In this study, we developed MiRTrans, a program to predict MiRNA targets by Trans-omics data. The trans-omics data includes miRNA sequencing, transcriptome sequencing and degradome sequencing (Figure 1). MiRTrans consists of three steps. First, the potential miRNA targets were collected by combining the non-redundant targets predicted by sequence-based tools psRNATarget (Dai and Zhao, 2011) and Tapir (Bonnet et al., 2010). Second, the targets were eliminated if the expressions of miRNAs and their targets are weakly correlated by lasso regression. The significance of regression coefficients was evaluated by Wald test and they were usually zero for irrelevant targets through L1-norm constrain. Third, degradome sequencing was utilized to capture the miRNA targets by examining the cleaved transcripts regulated by miRNAs. The second and third steps were parallel and were based on the independent data sources. Finally, the predicted targets from these two steps were combined by Fisher's combination test. The *p*-values were further rectified by Bonferroni correction to deal with multiple testing issue. We reported MiRTrans predictions on *Capsicum* spp. the only plant with trans-omics data which we have full access to.

Our results showed MiRTrans produced more functional miRNA targets comparing with sequence-based predictions, where the targets from the same miRNA were prone to be enriched in Gene Ontology, KEGG pathway, gene co-expression modules, or predicted gene families. MiRTrans identified 58 miRNA-target pairs with high confidence for 18 miRNAs families conserved in eudicots. Most of these targets were transcription factors; this lent support to the role of miRNA as a key regulator in pepper. Our results also showed that only a small proportion of miRNA targets were shared between the predictions of miRNA-transcript expression dependency and degradome sequencing, which indicated that the miRNA targets may be lost if they were predicted by single data source (Section Results).

## Methods

### The MiRTrans algorithm

**Figure 2:**
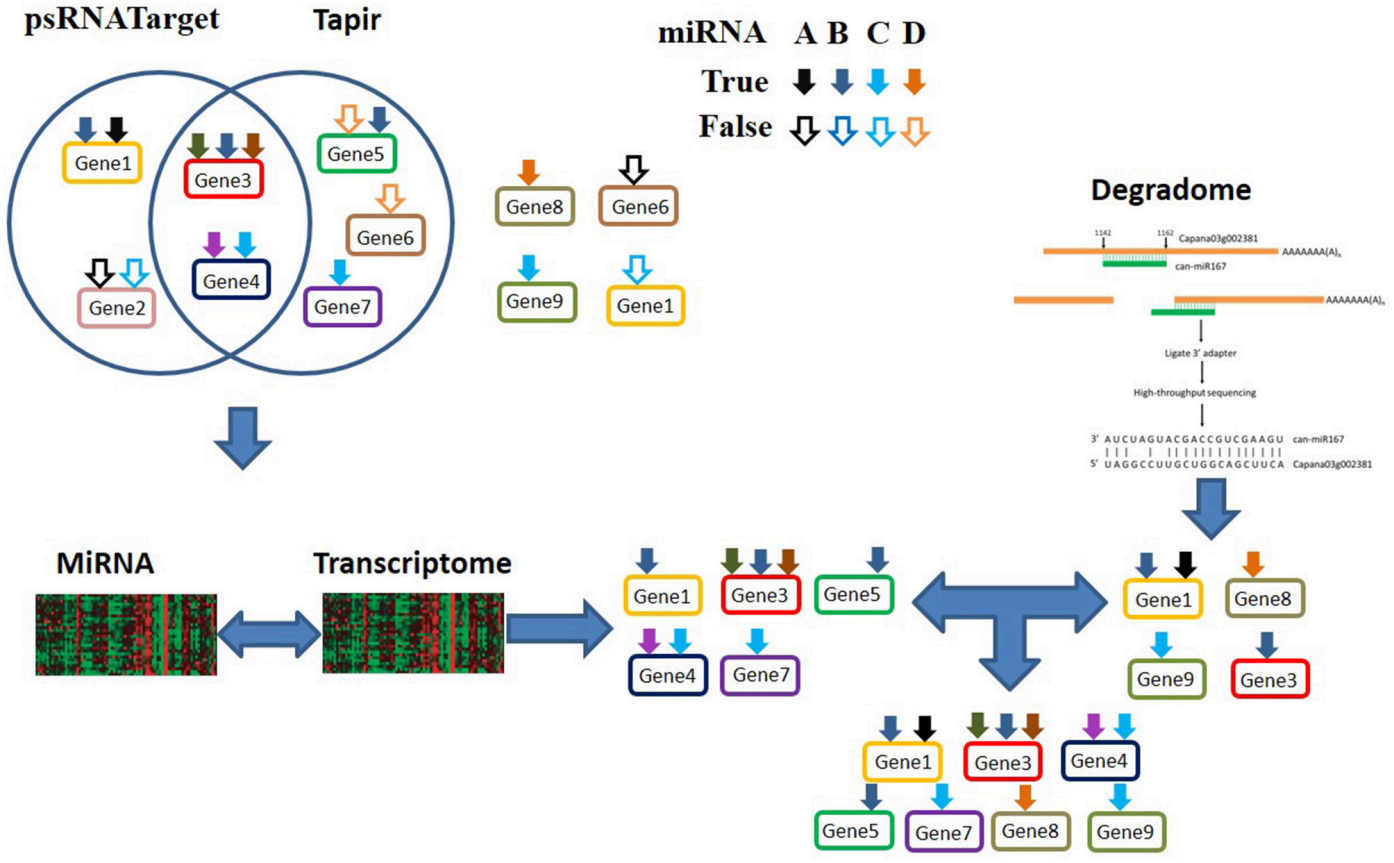
**An example for the miRNA targets prediction by MiRTrans**. Seven genes (Gene1 to Gene7) are assumed to be putative target genes of four miRNAs: A–D (demonstrated as black, dark blue, light blue and orange arrows), according to the non-redundant combination of psRNATarget and Tapir predictions. False positive miRNA-transcript pairs: A to Gene2, C to Gene2, D to Gene5, D to Gene6 are included the in sequence-based predictions. D to Gene8 and C to Gene9 are incorrectly missing in sequence-based predictions. By identifying the dependency between the expression of miRNAs and transcripts, MiRTrans refines the predictions by removing the false miRNA-transcript pairs from sequence-based predictions. Degradome sequencing data are incorporated to recoup those targets falsely removed by the previous steps (A to Gene1, B to Gene3, D to Gene8, C to Gene9).

The workflow of MiRTrans is described in Figure 1. To predict miRNA target more efficiently, MiRTrans incorporated the information from a variety of data sources including miRNA sequencing, transcriptome sequencing, and degradome sequencing. MiRTrans started from examining the characteristics of miRNA-transcript sequences by integrating the predictions from psRNATarget (Dai and Zhao, 2011) and Tapir (Bonnet et al., 2010). Next, lasso regression was applied to evaluate the expression dependency between miRNA-transcript pairs from sequence-based prediction. Weakly correlated pairs were considered to be less reliable and removed from initial pool (Section Methods). Parallelely, miRNA targets were also predicted by the cleaved transcripts which were thought as the productions of miRNA regulation (Section Methods). The two sets of miRNA targets were integrated and the *p*-values were calculated by Fisher's combination test followed by Bonferroni correction. Figure 2 illustrates an example on how MiRTrans works.

### Data preparation

#### MiRNA sequencing

The 17 samples for miRNA sequencing are derived from different development stages and tissues of *Capsicum* spp. The paired-end reads of five samples were available on Sequence Read Archive (SRP019257), and the other 12 sequenced samples were prepared for sequencing by Illumina HiSeq 2000.

Putative pre-miRNAs were inferred by aligning trimmed reads to the plant pre-miRNAs sequences in inmiRBase (Kozomara and Griffiths-Jones, 2014) by SOAP2 (Li et al., 2009). We removed the pre-miRNAs that failed in predicting miRNA secondary structure or positional overlaps and orientation of mature miRNA sequences within the respective stem-loop structure. The miRNA families were also limited if they were inconsistent with the confident miRNA annotation guidelines or current literature (Rhoades et al., 2002; Meyers et al., 2008; Hwang et al., 2013). We annotated 176 miRNAs with high confidence and their corresponding pre-miRNAs from 64 families (Table S1). The transcripts per million (TPM) was calculated for each miRNA followed by quantile normalization (Table S2).

#### Transcriptome sequencing

The matched 17 samples were prepared for transcriptome sequencing. Raw reads of 14 samples were available on Sequence Read Archive (SRP019256) and the other three samples were newly sequenced. The sequences of pepper transcripts were downloaded from The Pepper Genome Database (http://peppersequence.genomics.cn/page/species/download.jsp). The transcripts were predicted by *de novo* gene prediction, homology searching and transcriptome inference (Qin et al., 2014). These reads were mapped to pepper reference genome by TopHat (Trapnell et al., 2009) allowing at most five mismatches; the RPKM was calculated for each transcript followed by quantile normalization (Table S3).

#### Degradome sequencing

For degradome sequencing, we mixed equal amounts of five different tissues (flower, fruit, leaf, root, and stem) to capture the 5' ends of uncapped RNAs by using Illumina HiSeq 2000. CleaveLand classified miRNA hitting positions into four categories (Addo-Quaye et al., 2009). Category 0 and Category 1 were accorded on maximum depth and maximum value. If both maximum depth and maximum value were unique, these positions were labeled as Category 0. If multiple maximum depths and maximum values existed, these positions were labeled as Category 1. The positions with hit number larger than the median value and smaller than the maximum are labeled as Category 2. Other positions with more than one read coverage were labeled as Category 3. The positions covered by only one read were marked as Category 4. Targetfinder (Allen et al., 2005) aligned miRNAs to the cleaved transcripts of Category 0 or 1, followed by removing the alignments of distance more than 4.5 (Table S4).

### MiRNA target predictions

#### Sequence-based predictions

**Figure 3:**
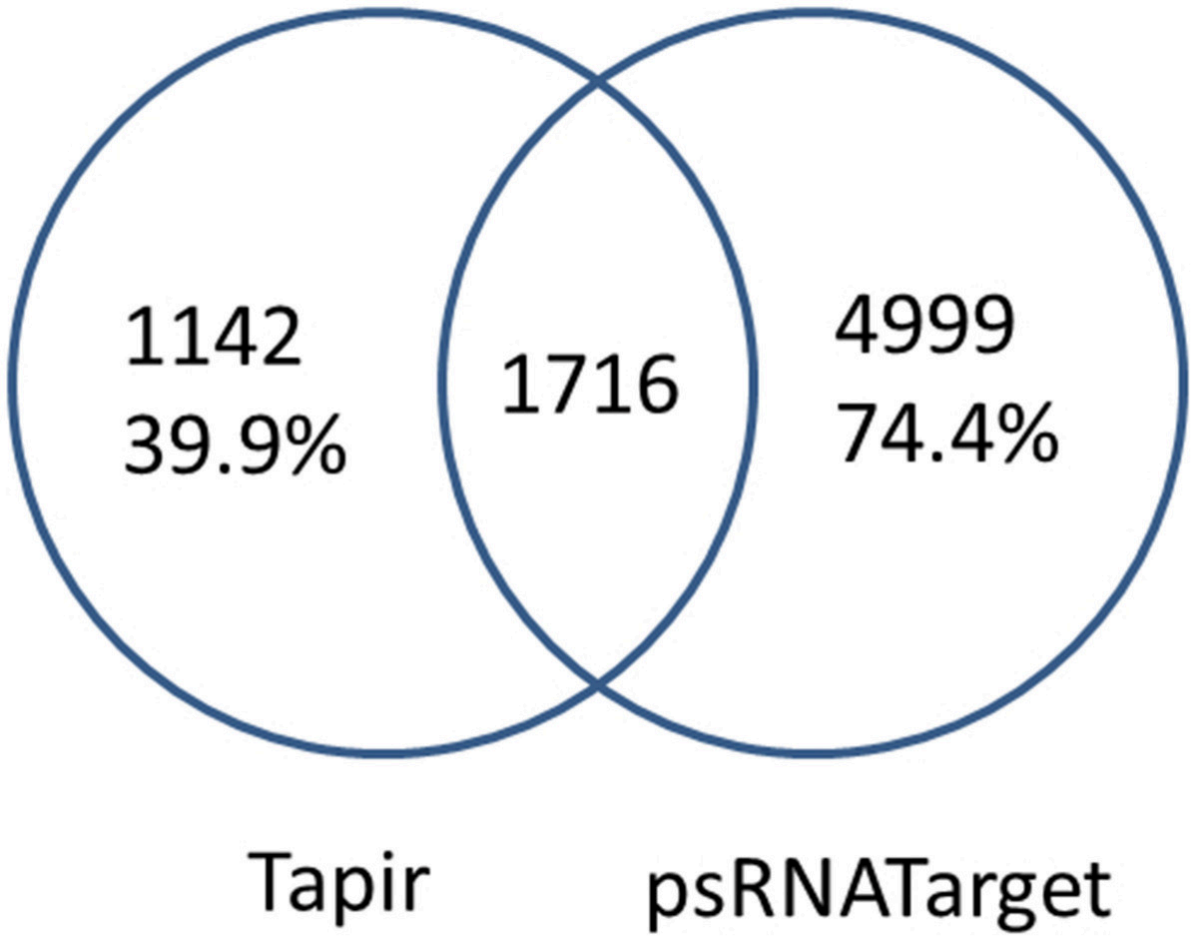
**MiRNA targets prediction by psRNATarget and Tapir**. Only 60.0% (Tapir) and 25.6% (psRNATarget) of the miRNA-transcript pairs are shared between Tapir and psRNATarget.

For each miRNA, the putative miRNA targets were generated by combining two sequence-based prediction tools designed for plants, psRNATarget and Tapir. Because of a high degree of complementarity was required for miRNA binding in plants, we expected these two programs to arrive at similar targets. However, that turned out not to be the case (Figure 3). To avoid missing any putative targets, we merged the non-redundant predictions from the two programs.

#### Lasso regression to determine expression dependency

Previous studies have shown sequence-based predictions are full of false positives. MiRTrans were eliminated these false positives by examining of the expression dependency between miRNAs and their targets. We assumed the expression of a particular transcript can be predicted by all the miRNAs that regulated it (Le et al., 2013). Lasso regression (Lu et al., 2011) was applied to alleviate over-fitting and remove irrelevant miRNAs. In lasso regression, the association between miRNAs and transcript was defined as “direct connection” by controlling the other miRNAs predicted to regulate the same transcript in sequence-based predictions.

For a transcript *i*(*i* = 1…*L*), its expression *y*^*i*^ was modeled by a simple linear regression model, including those potential miRNA $x_{k}^{i}\left(k\;=\;1\ldots P_{i}\right)$ inferred to regulate transcript *i* in sequence-based predictions.

$$\begin{array}{l} y^{i}=\beta_{0}^{i}+\overset{P_{i}}{\underset{k\;=\;1}{\sum }}\beta_{k}^{i}x_{k}^{i} \end{array}$$

To address the over-fitting issue caused by incorporating too many predictors, lasso regression was applied by introducing *L*_1_-norm penalty to make the regression coefficient $\beta_{k}^{i}$ to be zero if the regulation did not exist.

$$\begin{array}{l} y^{i}=\beta_{0}^{i}+\overset{P_{i}}{\underset{k\;=\;1}{\sum }}\beta_{k}^{i}x_{k}^{i}+\lambda \overset{P_{i}}{\underset{k\;=\;1}{\sum }}|\beta_{k}^{i}|\left(i\;=\;1\ldots L\right) \end{array}$$

$$\begin{array}{l} {\hat{\beta }}_{lasso}^{i}=\underset{\beta }{\mathrm{argmin}}\left\{\overset{N}{\underset{a\;=\;1}{\sum }}{\left(y_{a}^{i}-\beta_{0}^{i}-\overset{P_{i}}{\underset{b\;=\;1}{\sum }}\beta_{b}^{i}x_{a,b}^{i}\right)}^{2}\right\} \end{array}$$

$$\begin{array}{l} \text{subjectto}:\overset{j}{\underset{b\;=\;1}{\sum }}|\beta_{b}^{i}|\leq s \end{array}$$

$$\begin{array}{l} S>0 \end{array}$$

where *N* is the number of sequenced samples (17 in this study). lasso regression was adopted to restrict $\overset{P_{i}}{\underset{b\;=\;1}{\sum }}\beta_{b}^{i}$ by introducing the shrinkage parameter λ to control the sparsity of the regression model, which was determined by the minimum mean squared error from cross validation. There were no appropriate approximation approaches to evaluate the standard error of non-zero regression coefficients ($SE\left(\hat{\beta }\right)$) from lasso regression. Therefore, we performed 10,000 bootstraps to estimate $SE\left(\hat{\beta }\right)$ by fitting lasso regression with the same number of randomly selected miRNAs. The *p*-values to evaluate the expression dependency between miRNAs and the transcript were calculated by Wald test:

$$\begin{array}{l} \frac{\hat{\beta }-\beta_{null}}{SE\left(\hat{\beta }\right)} \end{array}$$

We assumed β_*null*_ = 0 for irrelevant miRNA and transcripts.

#### MiRNA target prediction by degradome sequencing

The potential transcript cleavage positions were determined by CleaveLand in conjunction with Targetfinder with default parameters. We chose the targets with alignment distance smaller than 4.5 and calculated the p-value as the likelihood of observing a degradome “peak” at the tenth nucleotide of the binding site. We adopted Fisher's combination test to incorporate the p-values from lasso regression and degradome sequencing followed by Bonferroni correction.

#### Validation approaches

Because of lacking gold standard to evaluate the performance of MiRTrans, it was validated by calculating the functional enrichment of predicted targets. We assumed the real targets of a miRNA should be engaged in similar biological processes, and hence should be enriched in the particular functional modules. We collected the functional modules from four functional sources: Gene Ontology, KEGG pathway, gene co-expression modules, and predicted gene families. The Gene Ontology annotations for genes were determined by their InterPro (Mitchell et al., 2015) entries. All transcripts were aligned against KEGG (Kanehisa et al., 2014) (Release 58) proteins to determine the pathways they were involved in. We applied OrthoMCL (Li et al., 2003) to define the gene family as a group of genes that were descended from the identical gene in the most recent common ancestor of the considered species.

WGCNA (Weighted Gene Co-expression Network Analysis) (Langfelder and Horvath, 2008) was applied to identify the co-expression network of 16,357 expressed transcripts from 17 development stages (transcripts with RPKM>1 for all stages). The correlation between two transcripts *x*_*i*_ and *x*_*j*_ was calculated by the absolute value of the Pearson coefficient (|*corr*(*x*_*i*_, *x*_*j*_)|). WGCNA constructed the similarity matrix for all transcript pairs, which is further transformed to a weighted adjacency matrix by introducing power function $a_{i,j}=|corr\left(x_{i},x_{j}\right){|}^{\beta }$, β = 12 was chosen to guarantee that the modules follow a scale-free topology. The total node connectivity of transcript *i* was defined as $k_{i}=\underset{j}{\sum }a_{ij}$. WGCNA proposed a “topology overlap” (TO) to define the correlation between two transcripts by averaging the adjacency information over all their network “neighbors.” The TO between transcripts *i* and *j* was calculated as $\omega_{ij}=\frac{l_{ij}+a_{ij}}{\text{min}\left\{k_{i},k_{j}\right\}}$, where $l_{ij}=\overset{L}{\underset{u\;=\;1}{\sum }}a_{iu}a_{ju}$ and it represented the degree of transcripts connection between *i* and *j*. The topological overlap matrix was composed by TO values and clustered by “dynamic tree cut” algorithm (Langfelder et al., 2008). The transcripts were clustered to co-expression modules according their topology overlap with the co-members in the same module.

## Results

### Sequence-based prediction

For sequence-based prediction, MiRTrans combined the non-redundant predictions from psRNATarget and Tapir (Figure 3), and assumed all the true miRNA-target pairs were included in the combined results. psRNATarget reported 6,715 pairs, 162 of which are found to have more than one potential binding sites in the same transcript. 76.5% of the targets were predicted as the cleavage effect of miRNA binding. Tapir created 2,858 miRNA-target pairs, of which merely 1,716 (60.0%) are shared with psRNATarget. Finally, 7,857 miRNA-target pairs are collected by combining the predictions of psRNATarget and Tapir.

### The expression dependency between miRNAs and transcripts

MiRTrans eliminated the miRNA-transcript pairs with insignificant *p*-values (Section Methods, *p*-value > 6.36e–6 = 0.05/7,857 from Bonferroni correction), which were calculated from their regression coefficients by Wald test. After removing the irrelevant targets removal, MiRTrans kept 363 pairs (4.62% in sequence-based predictions) by considering their expression dependency.

### Degradome sequencing prediction

CleaveLand (Section Methods) was used to analyze the paired-end reads from degradome sequencing, which generated 586 miRNA-transcript pairs. The remaining 405 (69.1%) pairs, with *p*-values ≤ 0.05 and alignment distance <4.5, were selected for further analysis.

### Comparing MiRTrans with sequence-based predictions

**Figure 4:**
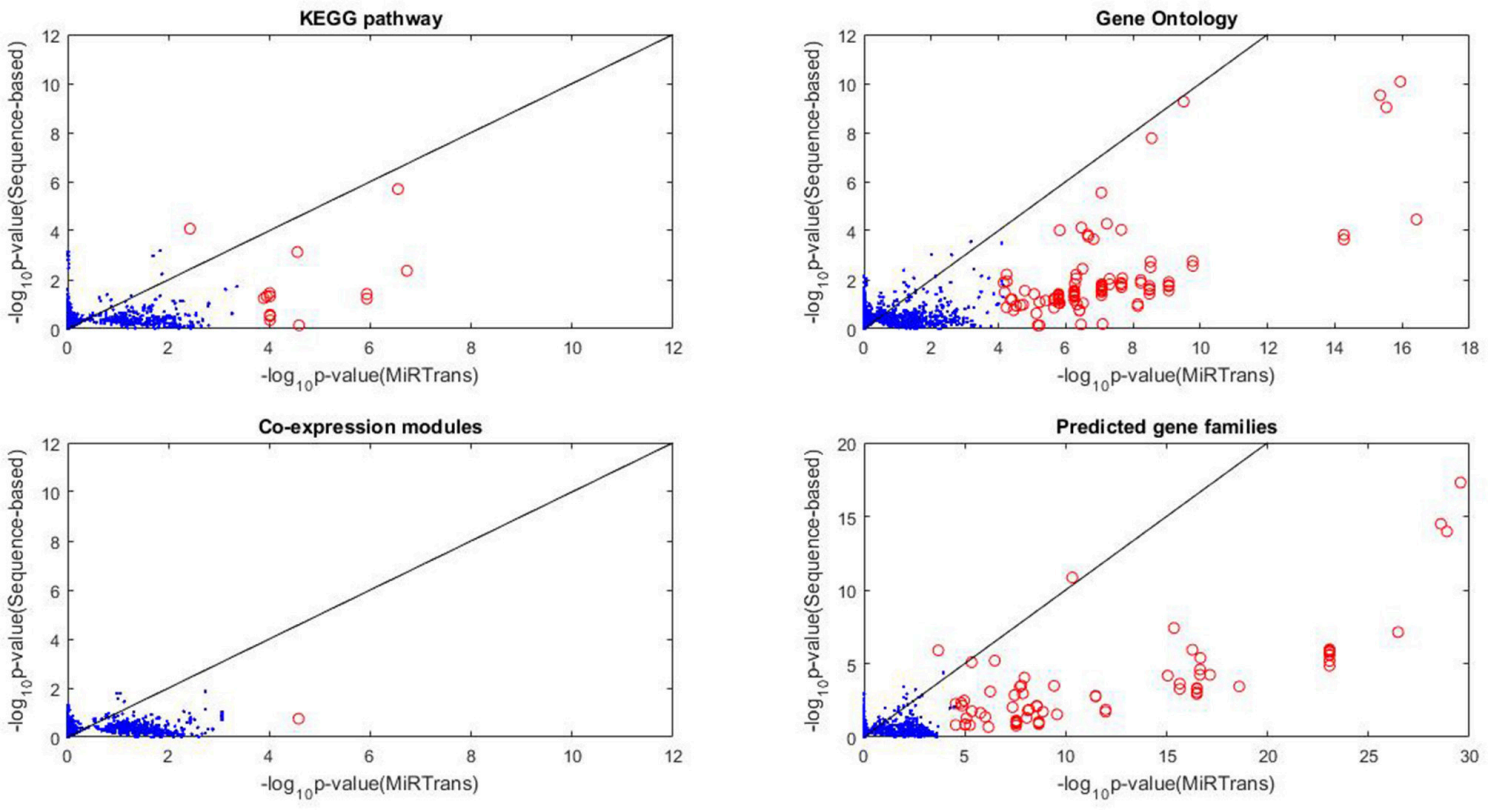
**Functional enrichment comparison between MiRTrans and sequence-based predictions**. MiRTrans achieved more functional miRNA targets than the sequence-based predictions.

MiRTrans produced 774 miRNA-transcript pairs by integrating the targets predicted by their expression dependencies and cleaved transcripts. To compare with sequence-based predictions, for each miRNA we generated 10,000 random networks, each including a miRNA ℜ and *k* targets ofℜ chosen from sequence-based predictions, where *k* was the number of targets of ℜ by MiRTrans. The functional enrichment from a variety of resources (KEGG pathway, Gene Ontology, Gene co-expression, and Predicted gene families) were applied to evaluate the performance of target prediction. We chose the functional modules with ≥ five genes from the four resources to avoid misleading significance. This resulted in 2,762 functional modules, including 275 KEGG pathways, 689 Gene Ontology terms, 112 gene co-expression modules and 1,686 predicted gene families. For the targets of each miRNA, the enrichment scores of the targets from MiRTrans were compared to the average value of 10,000 random networks. The significant functional modules (with *p*-value < $\frac{0.05}{\text{the number of modules}}$, red circle in Figure 4) were illustrated to compare the predicted targets between MiRTrans and sequence-based predictions.

Comparing with sequence-based predictions, MiRTrans produced more functional miRNA targets (*p*-value = 8.2252e-33 for predicted gene families, *p*-value = 7.8417e–32 for gene co-expression modules, and *p*-value = 3.7142e–15 for KEGG pathway). Furthermore, no significant difference was observed for Gene Ontology (*p*-value = 0.8173), because more sequence-based random networks were with marginal *p*-values. Nevertheless, more modules with significant *p*-values were prone to be included in MiRTrans predictions (Figure 4).

**Figure 5:**
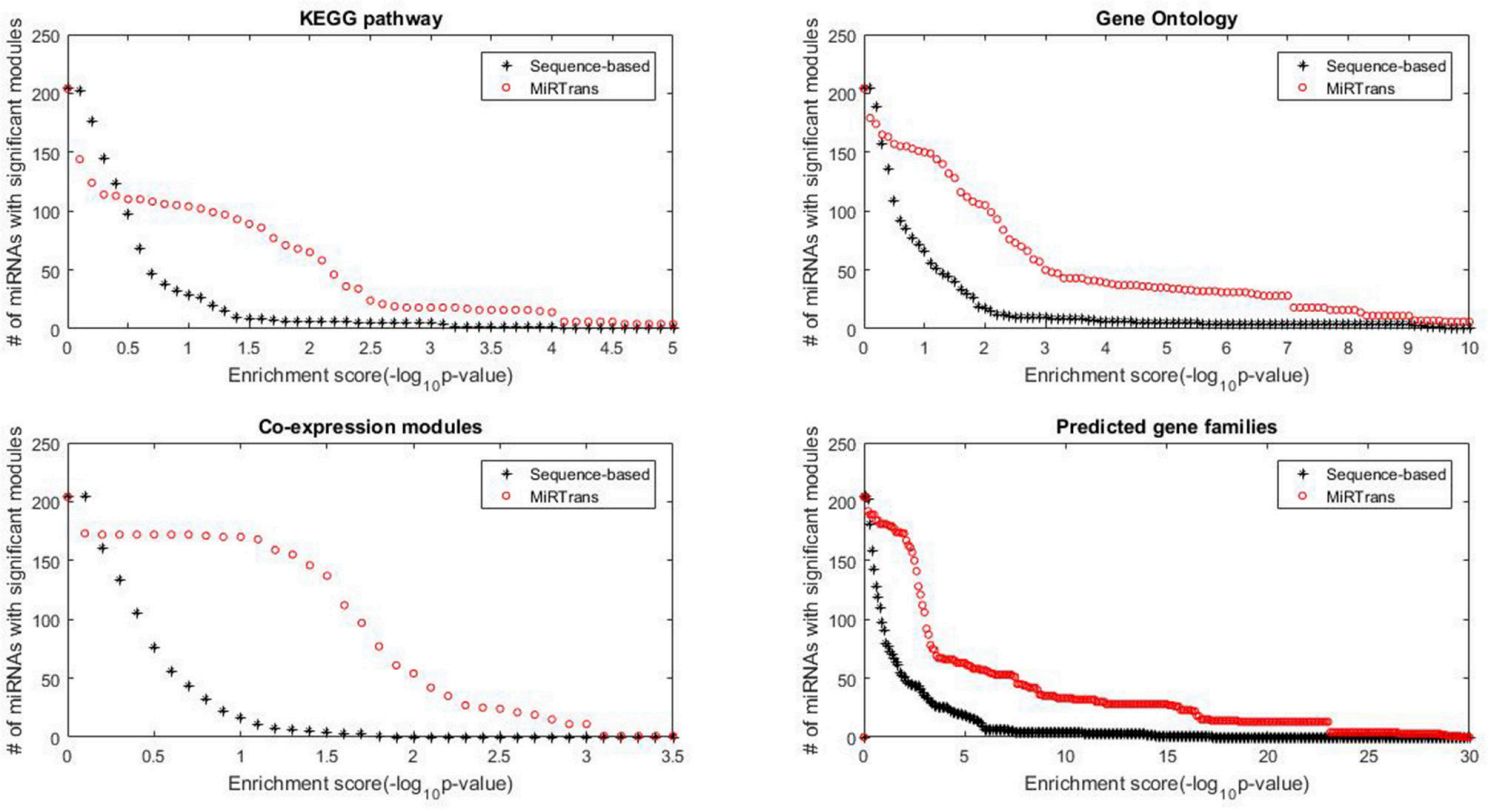
**The absolute number of miRNAs, whose targets are enriched in at least one significant function module for MiRTrans and sequence-based predictions**.

**Figure 6:**
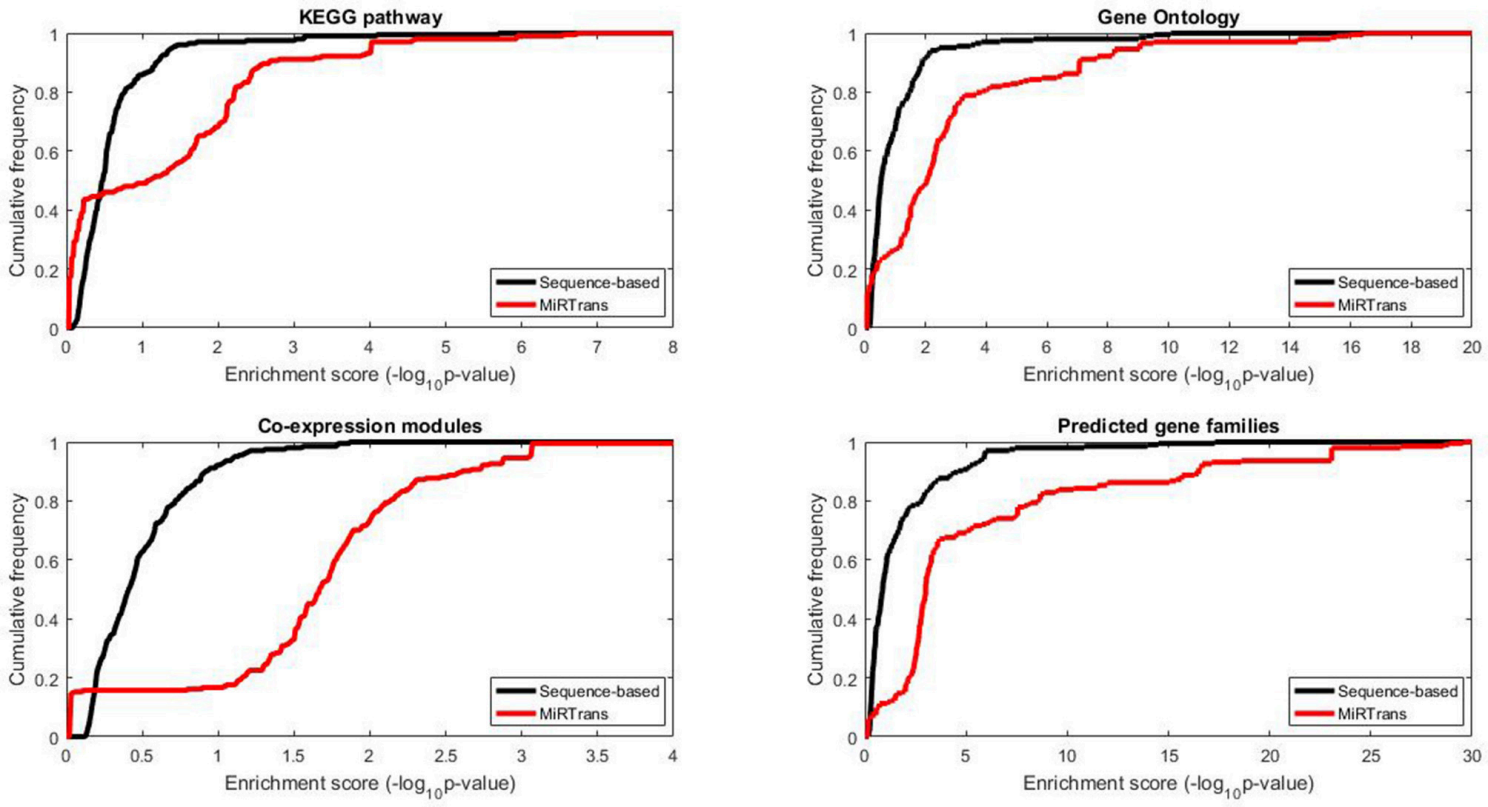
**The cumulative frequency of miRNAs, whose targets are enriched in at least one significant function module for MiRTrans and sequence-based predictions**.

The predicted targets were also evaluated by the capability to infer the miRNA functions. We performed two experiments to compare the capability for predicting miRNA functions between MiRTrans and sequence-based predictions. In Figures 5, 6, we investigated the absolute number and the cumulative frequency of miRNAs, whose targets were significantly enriched in functional modules by an increment of *p*-value thresholds. Compared to sequence-based predications, more miRNAs were annotated by the functions of their targets involved in. The targets of a miRNA were more likely to be from the same gene family, not only because of their similar sequence characteristics, but also they participated in the same synthesis or growth procedure (Song et al., 2011). Using a *p*-value threshold of 1e–4, MiRTrans predicted the functions of 22% of total miRNAs, whereas sequence-based predictions achieved a rate of mere 3%. We cannot readily identify miRNA functions by co-expression modules or signaling pathways because they were usually regulated by multiple miRNAs.

It is interesting to distinguish that how many contributions of the targets predicted by expression dependency and degradome sequencing. We calculated the functional enrichment for the targets predicted by expression dependency and degradome sequencing, respectively. In Table 1, the *p*-values were calculated by Wilcoxon-Mann-Whitney test against sequence-based predictions. The targets predicted by expression dependency and degradome sequencing demonstrated consistent trend of functional enrichment even they only shared two miRNA-target pairs. This phenomenon suggested single data source was not enough to capture all the miRNA targets.

**Table 1:** **Comparison of functional enrichment *p*-values of miRNA targets predicted by expression dependency and degradome sequencing**.

	**KEGG pathway**	**Gene Ontology**	**Co-expression**	**Predicted gene families**
Expression dependency	6.3750e-11	0.1736	4.9417e-21	8.9634e-45
Degradome sequencing	5.2916e-10	0.5368	1.5037e-18	5.2197e-20
MiRTrans	3.7142e-15	0.8173	7.4817e-32	8.2252e-33

*P-values were calculated by Wilcoxon-Mann-Whitney test. The miRNA targets predicted by either expression dependency or degradome sequencing were effective, but they were complementary with each other*.

### Identification and classification of the targets for pepper miRNAs

In our previous study, we reported 176 annotated miRNAs for *Capsicum* spp (Table S5) (Qin et al., 2014). In this study, after removing the transcripts shorter than 150 bp, MiRTrans revealed 186 targets of 18 conserved miRNA families and 68 targets of 15 pepper-specific miRNA families (Tables S6, S7). Additionally, there were 103 genes targeted by 26 pepper novel miRNA families (Tables S6, S7). Most of the targets of a conserved miRNA have similar sequence characteristics and belonging to the same gene family; this has been observed in the previous findings (Jones-Rhoades et al., 2006; Chen, 2009). We observed six conserved miRNAs, namely can-miR156, can-miR162, can-miR164, can-miR169, can-miR171, and can-miR172 with at least 10 targets, while other pepper-specific miRNAs appeared to have fewer targets except can-miR482, can-miR2873, and can-miR6149 (Tables S6, S7). We identified 18 out of 59 miRNA families binding to transcription factors; most of them were auxin response factors, TCP family transcription factors and NAC transcription factors (Table S7). These findings indicated the roles of these miRNA families in post-transcriptional regulation and transcriptional networks. Besides transcription factors, other identified targets were involved in macromolecule metabolic process, regulation of metabolic process, and nucleic acid binding etc. (Table S8).

### The comparison of miRNA targets between pepper and other plants

Several miRNA-transcript pairs predicted by MiRTrans were conserved across many plants, such as *Arabidopsis*, rice, soybean, and maize. Many predicted miRNA targets encoded regulatory proteins (Tables S7, S9), suggesting that miRNA served as a kind of master regulator in plant (Jones-Rhoades et al., 2006). MiRTrans predicted 58 conserved targets in pepper for 18 highly conserved miRNA families in eudicots (*Arabidopsis*, rice, maize, soybean, and pepper) with high confidence (Tables S9, S10). There were 46 targets of them that encoded transcription factors, which may play an important role in gene regulatory networks (Jones-Rhoades et al., 2006).

In addition, we retrieved putative orthologs of pepper miRNA targets based on the information from the EnsemblCompara gene trees (Vilella et al., 2009) on peppersequence.genomics.cn and gramene.org (Liang et al., 2008). We revealed 33 orthologs in *Arabidopsis*, 22 orthologs in soybean, 25 orthologs in rice, and 19 orthologs in maize (Table S7). Most of these orthologs were transcription factors and were known to regulate plant development (Table S9) (Jones-Rhoades et al., 2006). The miR156 family targeted SBP proteins and played a critical role in regulating phase change and floral induction (Kasschau et al., 2003; Chen et al., 2004; Vazquez et al., 2004; Allen et al., 2005; Wu and Poethig, 2006; Addo-Quaye et al., 2008; Wu et al., 2009). Previous studies reported that miR172 bound to a set of AP2 transcription factors (Addo-Quaye et al., 2008; Li et al., 2010; Song et al., 2011; Lee et al., 2014; Liu et al., 2014; Wang et al., 2014) (Table S9), but presented a variety of functions in different plants: Controlled genes related to flowering time and floral organ in *Arabidopsis* (Aukerman and Sakai, 2003; Kasschau et al., 2003; Jung et al., 2007; Mathieu et al., 2009; Wang et al., 2009; Wu et al., 2009); regulated inflorescence development in maize (Chuck et al., 2007a,b); involved in the regulation of vegetative and reproductive branching in rice (Zhu et al., 2009; Lee et al., 2014; Wang et al., 2015). The shared miRNA targets among *Arabidopsis*, maize, rice, and pepper suggested the existence of a similar mechanism of phase change and flowering time control. Similarly, miR159 regulated *MYB* genes and had various roles in flower development of *Arabidopsis* (Palatnik et al., 2003; Achard et al., 2004; Millar and Gubler, 2005; Alonso-Peral et al., 2010) and rice (Li et al., 2010), while miR319 controlled floral organ size and shape in *Arabidopsis* (Palatnik et al., 2003; Nag et al., 2009). Our predictions were consistent with previous studies of miR164, which regulated *NAC* genes that function in organ boundary formation (Laufs et al., 2004; Guo et al., 2005; Peaucelle et al., 2007; Sieber et al., 2007) (Table S9). In those non-transcription factor targets (Table S10), *DCL1* and *AGO1*, which were predicted as miR162 and miR168 targets, played a key role in tuning plant biogenesis and function (Xie et al., 2003; Vaucheret et al., 2004; Vazquez et al., 2004).

## Discussion

We presented MiRTrans, a trans-omics based program, to infer miRNA targets from three data sources: miRNA sequencing, transcriptome sequencing, and degradome sequencing. To our best knowledge, this is the first investigation to analyze and compare the contributions of different available technologies and trans-omics data sources for predicting miRNA targets. MiRTrans extracted and utilized three types of information: First, sequence characteristics, such as miRNA-target sequence complementarity, the sequence hybridization energy, and target site multiplicity; second, the expression dependency between miRNA and transcript; third, the cleaved transcripts were detected by sequencing the 5' ends of uncapped RNAs. These cleaved transcripts were assumed to be the productions of miRNA regulation. We further evaluated the performance of MiRTrans by comparing the targets functional enrichment with sequence-based predictions. These results supported that MiRTrans can generate more functional relevant miRNA targets than sequence-based predictions. The source code of MiRTrans is publicly available on https://github.com/zhanglu295/MiRTrans.

Degradome sequencing, designed to capture cleaved transcripts, has been widely applied in plants to predict miRNA targets. Successful stories were reported in *Arabidopsis* (Addo-Quaye et al., 2008), rice (Li et al., 2010), soybean (Song et al., 2011), and apple (Xia et al., 2012), it has yet to be proved whether all the targets were captured. There are three limitations of degradome sequencing in miRNA target prediction. First, RISC-mediated transcript cleavage cannot explain all the miRNA regulation in plant. The miRNAs regulation is via two molecular mechanisms: transcriptional degradation and translational repression (Jones-Rhoades et al., 2006; Gandikota et al., 2007). Degradome sequencing can only capture those cleaved transcripts rather than those repressed proteins. For example, miR172 regulates flowering time and floral organ by repressing the protein of *APETALA2* (*AP2*), rather than degrade its transcripts (Aukerman and Sakai, 2003; Chen, 2004; Schwab et al., 2005). Second, the sample for degradome sequencing were collected from mixed tissues in this study, the transcripts with low expression may be overwhelmed in the highly expressed transcripts; third, CleaveLand is the most famous program to analyze degradome sequencing data, but it applies Targetfinder to determine which miRNAs bind to the cleaved transcripts. Targetfinder is a sequence-based miRNA target prediction program for plant, and cannot avoid sequence supplementarity by random chance.

The expression dependency between miRNA and transcript is an indirect evidence in target prediction and is easily influenced by gene co-expression and miRNA temporospatial specificity. The transcripts may be incorrectly predicted as miRNA targets, if they are observed highly co-expressing with the real targets. miRNA regulation is unlikely to occur anytime and anywhere, which results in the unstable trend of expression correlations. Degradome sequencing alleviates these issues by directly sequencing the cleaved transcripts from mixed samples. Comparing the targets predicted by degradome sequencing and expression dependency, we found most of them were unique and had the same trend in functional enrichment, suggesting these two data sources were orthogonal. MiRTrans reduced the number of miRNA-target pairs from 7,857 to 774 (9.85%) and a significant reduction (from 148 to 20) of average targets for each miRNA from sequence-based predictions. The targets predicted by MiRTrans are more likely to be involved in the same predefined functional modules than those from sequence-based predictions.

The availability of next generation sequencing provides us an unprecedented opportunity to predict miRNA targets from trans-omics data. In this paper, we introduced MiRTrans, a trans-omics based program, that leveraged miRNA sequencing, transcriptome sequencing and degradome sequencing to predict miRNA targets. MiRTrans generated an atlas of the miRNA targets for pepper. Some of these targets have orthologs in other plants such as *Arabidopsis*, rice, maize, and soybean as validated by RLM-5' RACE, degradome, and/or miRNA-resistant/Agroinfiltration experiments. We discovered that data from different sources are orthogonal to each other, which suggested that previously reported miRNA targets that rely on a single data source may be incomplete.

## Author contributions

SL and KH supervised the work and together with LZ, CQ, and JM, developed MiRTrans and experiments. LZ, JM implemented the MiRTrans method, XC, ZW, XL, JC, and XT did the experiments on pepper. CQ and KH provided degradome sequencing data and other trans-omics data. LZ, CQ, and SL wrote the manuscript. All authors have read and approved the final manuscript.

## Funding

The work described in this paper was supported by a grant of GRF Project from RGC General Research Fund [9041901 (CityU 118413)], the Zunyi City Natural Science Foundation of China (No. 201201), the Guizhou Province and Zunyi City Science and Technology Cooperation Project of China (No. 201307, 201542), the Zunyi County Technology Cooperation Project (SSX201407), the National Natural Science Foundation of China (31372076), and Key Lab Construction Project of the Educational Department of Guizhou Province (Guizhou Education Cooperation KY [2014] 212).

### Conflict of interest statement

The authors declare that the research was conducted in the absence of any commercial or financial relationships that could be construed as a potential conflict of interest.
